# Effect of Fermented Soybean (FSB) Supplementation on Gastroesophageal Reflux Disease (GERD)

**DOI:** 10.3390/nu16162779

**Published:** 2024-08-20

**Authors:** Eugenie Sin Sing Tan, Rahela Zaman, Muhammad Akbar Memon, Chung Keat Tan

**Affiliations:** 1 Faculty of Medicine and Health Science, UCSI University, Kuala Lumpur 56000, Malaysia; eugenietan@ucsiuniversity.edu.my (E.S.S.T.); rahela@ucsiuniversity.edu.my (R.Z.); 2 Faculty of Medicine and Allied Medical Sciences, Isra University, New Hala-Mirpur Khas Rd Link, Hyderabad 71000, Pakistan; akbar_physician@yahoo.com

**Keywords:** fermented soybean (FSB), gastroesophageal reflux disease (GERD), reflux disease questionnaire (RDQ), inflammatory markers, Quality of Life in Reflux and Dyspepsia (QOLRAD)

## Abstract

Gastroesophageal reflux disease (GERD) is a prevalent chronic condition affecting the well-being of both adults and children in general medical practice. Research on the effects of fermented soybean (SB) supplementation in managing GERD is relatively new, with limited studies available. The existing research often lacks sufficient dosing regimens and study durations to differentiate between transient placebo effects and sustained benefits. In this study, the beneficial effects of FSB supplementation were investigated in 110 voluntary participants (NCT06524271). The participants were required to take 1 g of FSB supplement once daily for 12 weeks. GERD symptoms were evaluated using the Reflux Disease Questionnaire (RDQ), while inflammatory markers, including interleukin-4 (IL-4), interleukin-6 (IL-6), and interleukin-8 (IL-8), were measured to assess inflammation. The Quality of Life in Reflux and Dyspepsia (QOLRAD) questionnaire was used to evaluate participants’ quality of life. The results indicated that FSB supplementation significantly (*p* < 0.05) alleviated heartburn and regurgitation symptoms and reduced levels of IL-4, IL-6, and IL-8, indicating a notable anti-inflammatory effect. Additionally, significant (*p* < 0.05) improvements were observed in QOLRAD scores, particularly in vitality, emotional distress, and physical/social functioning. Collectively, our findings support the use of FSB as an adjuvant approach in managing GERD, with notable improvements in patients’ quality of life.

## 1. Introduction

Gastroesophageal reflux disease (GERD) is a prevalent and chronic condition in general medical practice, affecting adults and children [1,2]. According to the United Nations’ 2017 Revision of World Population Prospects, approximately 1.03 billion individuals suffer from GERD globally [2]. The global pooled prevalence of GERD was estimated to be 13.98% [2]. However, this figure varies significantly between regions and countries, with a higher GERD burden in less developed countries [3]. Moreover, the prevalence of GERD is on the rise globally, with an increase of 77.53% in cases from 1990 to 2019, underscoring the importance of managing this condition through effective healthcare strategies and preventive measures [3,4].

GERD happens when gastric contents reflux into the oesophagus, leading to troublesome symptoms, with or without complications. The most commonly reported symptoms are heartburn and regurgitation [5]. Some GERD cases could lead to additional complications, such as unexplained chest pain, cough, sore throat, frequent throat clearing, and asthma [1]. Furthermore, GERD profoundly impacts the quality of life (QoL) of affected patients, leading to work absences, hindering daily activities, impairing social interactions, disrupting sleep, increasing daytime sleepiness, reducing mealtime enjoyment, and decreasing workplace productivity [6,7,8,9]. Patients lose approximately 10.5 h of work per week due to GERD [10]. The QoL impairment is more pronounced in patients experiencing moderate to severe symptoms, with those having severe heartburn reporting the lowest QoL scores [7]. The QoL of GERD patients is comparable to individuals with other chronic diseases, such as diabetes, arthritis, or chronic heart failure [9]. In clinical practice, gastrointestinal QoL assessments are essential for comprehensive evaluations comparing patient cohorts and tailoring treatments to optimize outcomes [11].

Soy-based foods can enhance gastrointestinal (GI) health by modifying the composition and metabolic activity of the intestinal microbiome [12]. In particular, fermented soybean (FSB) showed more consistent benefits, due to the alteration of their physiochemical properties and enhancement of organoleptic characteristics, improving their nutritional and functional value [13]. FSB, especially those fermented by Lactobacillus delbrueckii, produce a postbiotic product effective in reducing heartburn severity and frequency, with efficacy comparable to a pharmacological placebo, maltodextrin [14]. These effects can be attributed to its bioactive properties, which can modulate gut microbiota, enhance the intestinal barrier, promote intestinal stem cell expansion, and suppress interleukin-6 (IL-6) levels [15,16]. It was also found to significantly reduce interleukin-8 (IL-8) and tumor necrosis factor-alpha (TNF-α) in cultured intestinal cells [17]. It is postulated that decreasing these pro-inflammatory cytokines will reduce epithelial cell inflammation over time. Notably, this supplementation significantly increases adherence rates and participant retention and improves their heartburn-related quality of life [14,18].

The current management of GERD symptoms involves proton pump inhibitors (PPIs), antacids, and H2-receptor antagonists. However, these pharmacological agents present specific limitations [19,20,21]. These limitations drive research into non-pharmacological treatments from natural sources. Dietary supplements have gained significant attention recently, due to their sustainable safety in long-term consumption [22]. They are increasingly recognized as a preventive measure or adjuvant therapy for mild to moderate disease conditions [23,24,25,26]. The research on the supplementation of FSB is relatively novel and limited in scope. The current studies often lack sufficient dosing regimens and study durations, which are essential to distinguish between transient placebo effects and the true long-term benefits of the supplementation [14]. Short-term studies typically capture immediate placebo responses influenced by participants’ expectations, which can obscure the actual therapeutic effects of the supplementation [27]. Therefore, this study aimed to determine the efficacy of FSB supplementation in improving GERD symptoms, suppressing inflammatory markers, and enhancing the overall quality of life. This was achieved through a rigorous three-month supplementation protocol coupled with four study visits to ensure comprehensive and reliable outcomes.

## 2. Materials and Methods

### 2.1. Participant Recruitment

**Figure 1:**
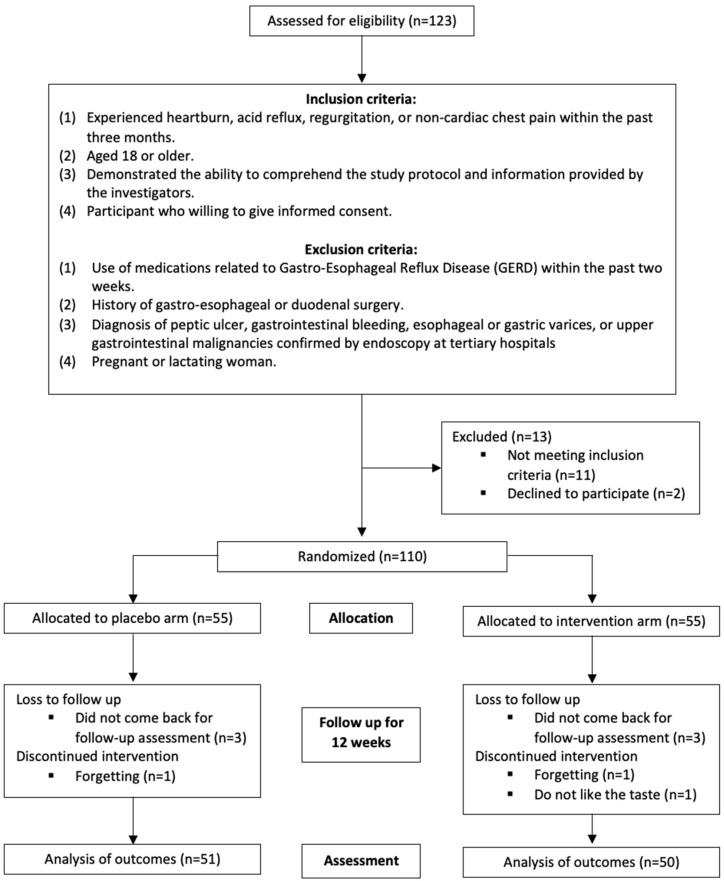
CONSORT protocol for the study described with flow diagram.

This randomized, double-blind, placebo-controlled study involved three months of supplementation. A total of 110 participants were enrolled, meeting the following inclusion criteria: (1) experienced heartburn, acid reflux, regurgitation, or non-cardiac chest pain within the past three months; (2) aged 18 years or older; (3) demonstrated the ability to comprehend the study protocol and information provided by the investigators; and (4) willing to give informed consent. Exclusion criteria included: (1) use of medications related to GERD, such as acid inhibitors, antacids, prokinetics, gastric mucosal protectors, herbs, probiotics, or related preparations within the past two weeks; (2) a history of gastroesophageal or duodenal surgery; (3) diagnosis of peptic ulcer, gastrointestinal bleeding, esophageal or gastric varices, or upper GI malignancies confirmed by endoscopy at tertiary hospitals; and (4) pregnancy or lactation (Figure 1). The study was registered in Clinicaltrials.gov (accessed on 29 July 2024) with the ID NCT06524271.

All participants received a participant information sheet and underwent a thorough explanation from investigators. Written informed consent was obtained from each participant. Randomization into the control or intervention group was conducted using a dice-rolling system. To maintain blinding, the placebo supplements administered were identical in appearance to the intervention supplements. Investigators were blinded to the participants’ identities. Participant’s compliance was monitored using a compliance form. This study adhered to the Declaration of Helsinki principles and the Malaysian Guidelines for Good Clinical Practice. Approval was obtained from the UCSI Institutional Ethics Committee (IEC).

### 2.2. Supplementation

The intervention supplement composed of *Lactobacillus delbrueckii* FSB, hydrogenated palm kernel oil, sodium caseinate, isolated soy protein, brown rice, inulin, digestive enzymes blend (lactase, protease, cellulase, lipase, amylase, pectinase, papain, and bromelain), galacto-oligosaccharides (GOS), and *Bifidobacterium longum*, powder form packaged in individual sachets (Nattome^TM^, Nattome, Kuala Lumpur, Malaysia). The dosage of FSB in each sachet was 1 g. The placebo supplement had the exact composition of ingredients but excluding FSB. Participants prepared the beverages by mixing the contents of each sachet with 150 mL of lukewarm water and consumed them once daily before meals. All supplements were labeled in a blinded manner. Participants were instructed to consume the supplement for 12 weeks without altering their habitual dietary or physical activity habits.

### 2.3. Instruments (Participant-Reported Outcomes)

At the baseline visit, participants’ demographic characteristics and anthropometric data were collected. Three follow-up visits were conducted at four-week intervals. Data on vital signs were recorded using a paper case report form (CRF). Blood pressure and heart rate were measured with an Omron automatic blood pressure monitor HEM 7120 (Omron Healthcare, Kyoto, Japan). The Reflux Disease Questionnaire (RDQ), which was psychometrically validated [28] and demonstrated utility in diagnosing GERD [29], was employed to evaluate GERD symptoms in the participants. The RDQ assessed six symptoms across three domains (heartburn, regurgitation, and upper abdominal pain) using a six-point Likert scale to measure frequency and severity over the preceding weeks. Each symptom was rated from 0 to 5, and the RDQ mean score was calculated by averaging responses to 12 items, resulting in scores ranging from 0 to 5. Higher scores indicated more severe and frequent symptoms.

The impact of GERD on quality of life was assessed using the Quality of Life in Reflux and Dyspepsia (QOLRAD) questionnaire. This questionnaire comprised 25 questions in five subdomains: emotional distress (questions 12, 14, 15, 17, 19, and 22), sleeping disorders (questions 8, 10, 11, 18, and 21), eating/drinking disorders (questions 3, 5, 9, 13, 16, and 20), physical/social function (questions 2, 6, 23, 24, and 25), and vitality (questions 1, 4, and 7). Participants rated each question on a seven-point Likert scale. The global score was calculated such that a lower score indicated a more severe impact on health-related quality of life. QOLRAD is a reliable, valid, and responsive instrument for assessing quality of life in subjects with gastrointestinal symptoms [30].

### 2.4. Laboratory Examination

Unstimulated saliva samples were collected using sterile 2.0-mL vials. Each participants uncapped the vial, placed a straw into the vial, and passively drooled down the straw for 90 s. All samples were assayed for different parameters in duplicates using respective kits. Interleukin-4 (IL-4) levels were assayed using Elabscience Human IL-4 ELISA Kit (Elabscience Biotechnology Co., Ltd., Houston, TX, USA), Interleukin-6 (IL-6) levels were assayed using Elabscience Human IL-6 ELISA Kit (Elabscience Biotechnology Co., Ltd., Houston, TX, USA), and Interleukin-8 (IL-8) levels were assayed using Elabscience Human IL-8 ELISA Kit (Elabscience Biotechnology Co., Ltd., Houston, TX, USA).

### 2.5. Statistical Analysis

The participant characteristics were presented as categorical data, expressed in frequencies and percentages. All outcomes were analyzed as continuous dependent variables and reported as mean ± SD. Changes in RDQ, QOLRAD, and laboratory outcomes from the baseline visit to the last follow-up visit were analyzed using a general linear model (GLM) for repeated measures. The within-subjects variable was defined as the sampling time point, while the grouping was tested as a between-subject factor. The homogeneity of variance and covariance structure of the dependent variables was assessed using Box’s M and Levene’s tests. The sphericity of residual covariance matrix was evaluated using Mauchly’s sphericity test. The results were considered significant if *p* < 0.05, with a 95% confidence interval. Statistical analysis was performed using SPSS version 29.0 (IBM Corp., New York, NY, USA) for MacOS.

## 3. Results

In total, 110 participants who had experienced heartburn, acid reflux, regurgitation, or non-cardiac chest pain in the past three months were recruited for the study. Of these, nine participants were excluded due to low compliance or loss to follow-up, resulting in a drop-out rate of 8.2% (Figure 1). Most participants were aged 36 to 45 years (n = 45, 44.6%), followed by those aged 46 to 55 years (n = 23, 22.8%) and 26 to 35 years (n = 16, 15.8%). There were more female participants (n = 65, 64.4%) than male participants (n = 36, 35.6%). Most participants had normal weight (n = 60, 59.4%), followed by overweight (n = 25, 24.8%), and lastly, underweight, and obese with 6.9% and 8.9%, respectively (Table 1). Participants’ blood pressures and heart rates were monitored throughout the study, and both vital signs showed very minimal changes between visits.

Heartburn subdomain scores in the intervention group decreased significantly from 0.420 ± 0.065 at the baseline visit to 0.220 ± 0.007 at the final follow-up visit, representing a 47.6% reduction, which was significantly (*p* < 0.01) higher than the reduction observed in the placebo group. A sub-analysis of the demographic factors showed that subjects with higher BMI responded better to supplementation. Similarly, the dyspepsia subdomain scores in the intervention group showed a significant reduction (*p* < 0.05) from 0.575 ± 0.093 to 0.340 ± 0.076, equivalent to a 40.9% decrease. Nonetheless, the reduction in the intervention group was not significantly different from the placebo group. Additionally, our findings indicated that the supplementation effectively suppressed regurgitation symptoms, reducing scores from 0.905 ± 0.073 to 0.335 ± 0.056, a 62.9% improvement, which was significantly better (*p* < 0.001) than the effects observed in the placebo group. A sub-analysis of the demographic factors showed that elderly subjects responded better to the supplementation (Table 2).

Participants’ changes in inflammation levels were assessed by measuring IL-4, IL-6, and IL-8 levels. In the intervention group, IL-4 levels decreased from 129.97 ± 37.73 pg/mL at baseline to 94.80 ± 24.32 pg/mL at the final visit, indicating a 27.1% reduction, which was significantly higher (*p* < 0.01) than the reduction observed in the placebo group. IL-6 levels followed a similar reduction from 4.407 ± 0.715 pg/mL to 2.494 ± 0.483 pg/mL during the consumption period, representing a 43.5% reduction and a significantly greater decrease (*p* < 0.05) than the placebo group. Similarly, reduction in IL-8 levels in the intervention group demonstrated a significantly (*p* < 0.05) higher reduction (19.8%) than placebo group, from 231.82 ± 49.67 pg/mL at baseline to 185.83 ± 19.76 pg/mL at the end of study (Table 3).

The overall scoring of QOLRAD improved in the intervention group, increasing from 5.268 ± 1.955 to 5.971 ± 1.999, indicating a 13.3% improvement, which was significantly higher (*p* < 0.05) than changes observed in the placebo group. However, a detailed examination of the subdomains revealed that improvements in the sleep disturbance subdomain (12.3%) and the food/drink problems subdomain (15.1%) were not significantly different from those in the placebo group. The vitality subdomain showed a 16.7% improvement, increasing from 5.027 ± 1.647 to 5.873 ± 2.010, significantly higher (*p* < 0.05) than the placebo group. The emotional distress subdomain improved from 5.287 ± 1.003 to 5.980 ± 1.036 by the end of the study, with a significantly higher improvement (13.3%, *p* < 0.05) compared to the placebo group. Lastly, the physical/social functioning subdomain demonstrated the most minor change (10.8%) among all the subdomains but still showed significantly higher improvement (*p* < 0.05) compared to the placebo group (Table 4).

## 4. Discussion

GERD is one of the most prevalent gastrointestinal disorders globally. A recent meta-analysis revealed that the prevalence of GERD varies widely across different countries and regions, ranging from 2.5% to 51.2%. This wide range underscores the influence of environmental, dietary, genetic, and stress-related factors on the incidence of GERD [31,32]. The pooled data from 102 studies spanning 37 countries provided a more precise global prevalence rate of 13.98%, with a specific prevalence of 9.7% reported in Malaysia [2,33,34]. This morbidity leads to a loss of quality of life and represents a substantial burden for national healthcare systems [35,36]. The pathophysiology of GERD is multifactorial, involving a complex interplay of genetic, physiological, and lifestyle factors [37]. Among the various risk factors for GERD, gender and age are two of the most significant. Research consistently demonstrated that GERD is more prevalent in women than in men. Specifically, conditions like functional heartburn and non-erosive reflux disease (NERD) are more common among women [38]. This higher incidence in women could be attributed to hormonal differences [39], the impact of pregnancy [40], and other gender-specific physiological factors. Our study corroborates this trend, showing a high prevalence of GERD among females. Also, this observation aligns with previous studies reporting a significant female predominance in GERD prevalence [41,42]. Age is another critical risk factor for GERD. Numerous studies have demonstrated that the incidence of GERD varies with age, often peaking during the productive years of adulthood [43,44]. This age-specific prevalence is likely due to a combination of factors, including changes in lifestyle, diet, and physiological functions that occur with aging [45,46,47]. Our study observed a high prevalence of GERD among participants aged 36 to 45 (n = 45, 44.6%) and those aged 26 to 35 (n = 16, 15.8%). Overall, these findings underscore the importance of considering gender and age when studying GERD and developing strategies for its prevention and management.

As described in epidemiological studies, GERD can be diagnosed based on common symptoms of heartburn and acid regurgitation [48]. Alarm symptoms (for example, dysphagia, weight loss, anaemia), atypical presentations (including chest pain and laryngeal symptoms) or lack of response to empiric therapy prompt further evaluation with an upper endoscopy [49]. To aid in the diagnosis of GERD, especially in primary care settings, the Reflux Disease Questionnaire (RDQ) was developed. It is a 12-item self-administered questionnaire to evaluate the frequency and severity of heartburn, regurgitation, and dyspeptic complaints. The RDQ is a practical tool for clinicians to identify GERD symptoms and assess their impact on patients’ daily lives [50]. The RDQ has been validated in multiple languages, ensuring its applicability and reliability across diverse populations worldwide. Its psychometric properties have been rigorously tested in primary care populations, demonstrating high internal consistency and reliability levels, with alpha coefficients ranging from 0.80 for the dyspepsia scale to 0.81 and 0.85 for the heartburn and regurgitation scales, respectively. Furthermore, the RDQ has shown strong test–retest reliability, with coefficients ranging from 0.80 to 0.88 [28]. A recent review of 116 studies has confirmed the reliability of the RDQ as an assessment tool, validating its use in evaluating the effectiveness of interventions in clinical trials. This comprehensive validation underscores the RDQ’s value in clinical practice and research settings, facilitating accurate diagnosis and monitoring of GERD progressions [51].

Heartburn is characterized by a burning sensation in the chest, which occurs when gastric acids travel back up the oesophagus, causing irritation to the lining. Our research has demonstrated that FSB supplementation for three months can effectively alleviate heartburn symptoms by a notable 47.6%. This significant improvement aligns with previous studies showing fermented soy supplementation can enhance the quality of life among adults suffering from heartburn [14]. The process of fermenting soy flour leads to the production of bioactive peptides, which are believed to possess modulatory effects on inflammation and could play a crucial role in reducing heartburn symptoms [52]. Specifically, studies have indicated that *Lactobacillus delbrueckii* subsp. *delbrueckii* R-187, a strain used in fermenting soybeans, can influence the production of cytokines in intestinal epithelial cells [17]. Furthermore, animal model studies have demonstrated that FSB can increase serum levels of prostaglandin E2, a compound known for its protective effects on the stomach lining. Additionally, it has also been shown to decrease the levels of inflammatory factors and reduce the expression of receptors related to gastric acid secretion in both gastric tissues and primary gastric parietal cells [53]. Sub-analysis revealed that subjects with a higher BMI responded more favorably to the supplementation in the improvement of heartburn symptoms. This finding is consistent with previous research suggesting that individuals with more severe conditions, such as obesity, may experience greater benefits from the treatment [54]. In contrast, while the symptoms of dyspepsia significantly improved by 40.9% during the study, no significant differences were observed between the placebo and intervention groups. This lack of differentiation could be attributed to the presence of digestive enzymes blend (lactase, protease, cellulase, lipase, amylase, pectinase, papain, bromelain) in both groups. Previous reviews suggested that digestive enzyme combinations have a modest response in ameliorating various symptoms of dyspepsia due to both functional and organic causes. Specifically, amylase, pepsin, and lipase are the main enzymes involved in the digestion of carbohydrates, proteins, and fats, respectively. Importantly, these enzyme combinations are considered safe and free from serious adverse effects [55]. Regurgitation, defined as the involuntary or voluntary expulsion of undigested food or liquid from the oesophagus or stomach back into the mouth, is another common symptom associated with GERD. Our study observed a significant 62.9% improvement in regurgitation symptoms in the intervention group, consistent with previous studies that demonstrated the effectiveness of soy products in reducing regurgitation, particularly in infants [56,57]. It is suggested that soy fiber may alter gastric physiology, leading to gastric emptying and reducing the likelihood of reflux [58]. Our findings also indicated that older subjects responded more positively to the supplementation, which may be attributed to the more severe presentation of GERD in this age group at the beginning of the study [59].

GERD-associated functional and structural abnormalities are caused by recurrent exposure of the oesophagus to acidic and nonacidic refluxate of gastric contents (containing duodenal and intestinal proteases as well as acid and gastric pepsin) from the stomach. Abnormal exposure of the oesophagus to gastric contents leads to chronic mucosal inflammation characterized by the release of IL-8, as well as other pro-inflammatory mediators, from the oesophageal mucosa [60]. Salivary biomarkers are novel, non-invasive approaches for diagnosing chronic inflammation, particularly in gastroesophageal conditions. Recent literature has delineated the normal levels of IL-4 at 15–25 pg/mL, IL-6 at 0.5–34 pg/mL, and IL-8 150–400 pg/mL [61]. However, it is crucial to recognize these reference values are subject to variation influenced by several factors, including the individual’s age, overall health status, and the specific laboratory methods employed for measurement. IL-8 plays a crucial role in the inflammation associated with various diseases of the upper gastrointestinal tract [62]. Expression of IL-8 was found to be correlated with erosive and non-erosive GERD [63,64]. Soybean peptides, derived from soybean hydrolysate, have demonstrated anti-inflammatory properties by reducing IL-8 mRNA expression in epithelial cells [65]. Moreover, soybeans fermented with lactic acid bacteria have shown greater efficacy in modulating IL-8 production [66], consistent with our findings of a 19.8% reduction in IL-8 levels following supplementation with FSB. Similarly, supplementation also demonstrated suppressive effects on IL-6 levels, with an overall 43.5% reduction during the study. IL-6, another pro-inflammatory marker, is abundantly present in the oesophageal mucosa of GERD patients; recurrent acid reflux is sufficient to stimulate oesophageal epithelial cells to produce IL-6, leading to esophagitis [67]. A recent epidemiological study conducted in Japan, where soy product is part of the main dietary intake, found an inverse association between total fermented soy food intake and IL-6 levels [68]. This effect is likely due to the high concentration of isoflavone and fibrinolytic enzymes in FSB that were found to possess anti-inflammatory properties, which can reduce the expression of IL-6 mRNA [69]. Similarly, IL-4, a pro-inflammatory cytokine, has been strongly correlated with GERD severity and frequency of acid reflux episodes [70,71,72]. Evidence indicates that FSB can alter the activities and expressions of various pro-inflammatory cytokines, including IL-4 [73], corroborated by our study, which reported a 27.1% reduction in IL-4 levels among participants in the intervention group. Further research on murine models also demonstrated the modulatory effects of FSB on Th2 lymphocytes, resulting in IL-4 suppression [74]. Based on these observations, therapeutic strategies targeting the modulation of IL-8, IL-6, and IL-4 production may offer effective treatments for GERD.

Quality of life and mental health is often associated with disease severity [75,76,77]. QOLRAD is a widely recognized questionnaire developed specifically to measure the impact of gastrointestinal diseases on patients’ physical, psychological, and social functions, which has been validated as a reliable assessment tool in a multinational clinical trial [78,79]. Notably, impairment of QOLRAD showed a strong correlation with patient-perceived severity and frequency of GERD symptoms [80]. Consequently, QOLRAD scores are frequently utilized to evaluate the efficacy of GERD management plans [81]. Our study observed a 13.3% overall improvement in QOLRAD scores after three months of supplementation, surpassing the minimally important change of 0.5 points necessary for a perceived beneficial treatment effect across all domains [82]. QOLRAD encompasses five subdomains: vitality, sleep disturbance, emotional distress, food/drink problems, and physical/social functioning. Our findings indicated statistically significant improvements in vitality (16.7%), emotional distress (13.3%), and physical/social functioning (10.8%) in the intervention group compared to the placebo group. These results align with previous studies demonstrating a high correlation between RDQ and QOLRAD scores [83,84]. A meta-analysis involving 1,485,268 participants from nine studies found that the risk of mental disorders was 2.57 times higher in individuals with GERD compared to controls [85]. Additionally, a separate review highlighted a correlation between anxiety and depression and the development of GERD [86]. Our study’s improvement in emotional distress may also be attributed to suppressed inflammation levels. Abnormal expression of inflammatory factors in GERD patients has been linked to central nervous system (CNS) inflammation, which can lead to emotional distress [87,88]. On the other hand, our results showed no additional effects of supplementation on food/drink problems and sleep disturbance scores, suggesting their association with dyspepsia symptoms. Dyspepsia is characterized by a complex of upper abdominal symptoms, including discomfort or pain, fullness, early satiety, abdominal distension, bloating, belching, and nausea, which can interfere with eating and drinking [89]. Furthermore, it is well established that sleep quality and duration are inversely correlated with dyspepsia occurrence, symptom frequency, and severity [90,91]. Overall, the observed improvements in the QOLRAD scores indicate that FSB supplementation effectively alleviates GERD symptoms.

This study is among the first globally to examine the effects of FSB in improving GERD conditions, employing a stringent RCT design and a rigorous three-month follow-up period. However, the authors would like to highlight that interleukin levels are sensitive to various factors and causes of inflammation. Future research should incorporate esophagogastroduodenoscopy (EGD) or pH monitoring with biopsy to validate the biomarker findings.

## 5. Conclusions

While FSB has demonstrated health-promoting benefits, the impact of lactic acid bacteria-fermented soybeans on gut health remains underexplored. The present work provides compelling evidence that FSB supplementation can significantly alleviate GERD symptoms, particularly heartburn and regurgitation, through its anti-inflammatory properties. These findings support using FSB as an adjuvant approach in managing GERD, with notable improvements in patients’ quality of life. Future research should further explore the underlying mechanisms of these effects and consider gender and age-specific strategies for GERD prevention and management.

## Figures and Tables

**Table 1:** Characteristics of Participant.

Characteristic	Frequency (%)
Gender (n/%)	
Male	36 (35.6)
Female	65 (64.4)
Age (years) (n/%)	
≤25	9 (8.9)
26–35	16 (15.8)
36–45	45 (44.6)
46–55	23 (22.8)
>55	8 (7.9)
Body Mass Index (BMI) (n/%)	
Underweight (<18.5)	7 (6.9)
Normal Weight (18.5–24.9)	60 (59.4)
Overweight (25–29.9)	25 (24.8)
Obese (≥30)	9 (8.9)

**Table 2:** Changes in Reflux Disease Questionnaire (RDQ) scores of participants during study, values are expressed as mean ± SD.

RDQ Domains	Group	Baseline	First Follow-Up	Second Follow-Up	Third Follow-Up	*p*-Value
Heartburn	Intervention	0.420 ± 0.065	0.445 ± 0.061	0.690 ± 0.028	0.220 ± 0.007	<0.01 *
	Placebo	0.490 ± 0.038	0.779 ± 0.045	0.603 ± 0.039	0.515 ± 0.051	
Dyspepsia	Intervention	0.575 ± 0.093	0.570 ± 0.087	0.750 ± 0.057	0.340 ± 0.076	0.466
	Placebo	0.564 ± 0.097	0.583 ± 0.093	0.613 ± 0.088	0.466 ± 0.016	
Regurgitation	Intervention	0.905 ± 0.073	0.785 ± 0.076	0.785 ± 0.059	0.335 ± 0.056	<0.001 *
	Placebo	0.897 ± 0.023	0.912 ± 0.042	0.373 ± 0.093	0.740 ± 0.017	

Statistically significant *p* values are marked in asterisks (*). *p*-value was calculated using general linear model (GLM) for repeated measures model

**Table 3:** Changes in inflammation levels of participant during study, values are expressed as mean ± SD.

Inflammation Levels	Group	Baseline	First Follow-Up	Second Follow-Up	Third Follow-Up	*p*-Value
Interleukin-4 (pg/mL)	Intervention	129.97 ± 37.73	108.73 ± 27.06	108.24 ± 10.49	94.80 ± 24.32	<0.01 *
	Placebo	137.29 ± 22.55	139.81 ± 26.57	171.20 ± 19.37	149.45 ± 33.68	
Interleukin-6 (pg/mL)	Intervention	4.407 ± 0.715	4.236 ± 1.138	5.012 ± 1.242	2.494 ± 0.483	<0.05 *
	Placebo	4.747 ± 1.246	3.418 ± 0.874	3.284 ± 0.592	4.982 ± 0.943	
Interleukin-8 (pg/mL)	Intervention	231.82 ± 49.67	223.12 ± 36.85	205.54 ± 26.19	185.83 ± 19.76	<0.05 *
	Placebo	209.78 ± 34.43	213.31 ± 22.21	206.27 ± 19.84	220.19 ± 25.66	

Statistically significant *p* values are marked in asterisks (*). *p*-value was calculated using general linear model (GLM) for repeated measures model.

**Table 4:** Changes in Quality Of Life in Reflux and Dyspepsia (QOLRAD) scores of participants during study, values are expressed as mean ± SD.

QOLRAD	Group	Baseline	First Follow-Up	Second Follow-Up	Third Follow-Up	*p*-Value
Overall	Intervention	5.268 ± 1.955	5.712 ± 1.675	5.642 ± 1.741	5.971 ± 1.999	<0.05 *
	Placebo	5.466 ± 1.850	5.621 ± 1.077	5.549 ± 1.799	5.512 ± 2.005	
Vitality	Intervention	5.027 ± 1.647	5.633 ± 1.024	5.567 ± 1.889	5.873 ± 2.010	<0.05 *
	Placebo	5.255 ± 1.647	5.536 ± 1.024	5.353 ± 1.889	5.405 ± 2.010	
Emotional Distress	Intervention	5.287 ± 1.003	5.730 ± 1.691	5.613 ± 1.785	5.980 ± 1.036	<0.05 *
	Placebo	5.484 ± 1.929	5.608 ± 1.153	5.578 ± 1.814	5.546 ± 2.032	
Sleep Disturbance	Intervention	5.340 ± 2.022	5.748 ± 1.741	5.612 ± 1.787	5.996 ± 1.061	0.535
	Placebo	5.509 ± 1.958	5.678 ± 1.148	5.580 ± 1.797	5.816 ± 2.025	
Food/Drink Problems	Intervention	5.143 ± 1.995	5.667 ± 1.670	5.670 ± 1.708	5.917 ± 1.987	0.339
	Placebo	5.135 ± 1.847	5.614 ± 1.979	5.477 ± 1.773	5.602 ± 1.975	
Physical/Social Functioning	Intervention	5.468 ± 1.101	5.780 ± 1.722	5.720 ± 1.797	6.060 ± 2.030	<0.05 *
	Placebo	5.565 ± 1.950	5.643 ± 1.142	5.690 ± 1.896	5.565 ± 2.068	

Statistically significant *p* values are marked in asterisks (*). *p*-value was calculated using general linear model (GLM) for repeated measures model.

## Data Availability

The datasets generated during and/or analyzed during the current study are available from the corresponding author on reasonable request. All of the individual participant data collected during the trial, after deidentification will be shared upon reasonable request. Additional documents including study protocol, statistical analysis plan, informed consent form and clinical study report will also be made available. The data will be available immediately following publication with no end date. Data will be shared with anyone who wishes to access with reasonable request. The data can be used for any types of analyses.
